# Supplementary material for: Super-resolution spectroscopic microscopy via photon localization
Source: Nat Commun. 2016 Jul 25;7:12290. doi: 10.1038/ncomms12290 (PMC4962472; doi:10.1038/ncomms12290)
Supplement: Supplementary Information — Supplementary figures 1-13, Supplementary notes 1-2, Supplementary References [file ncomms12290-s1.pdf]

Supplementary Figures

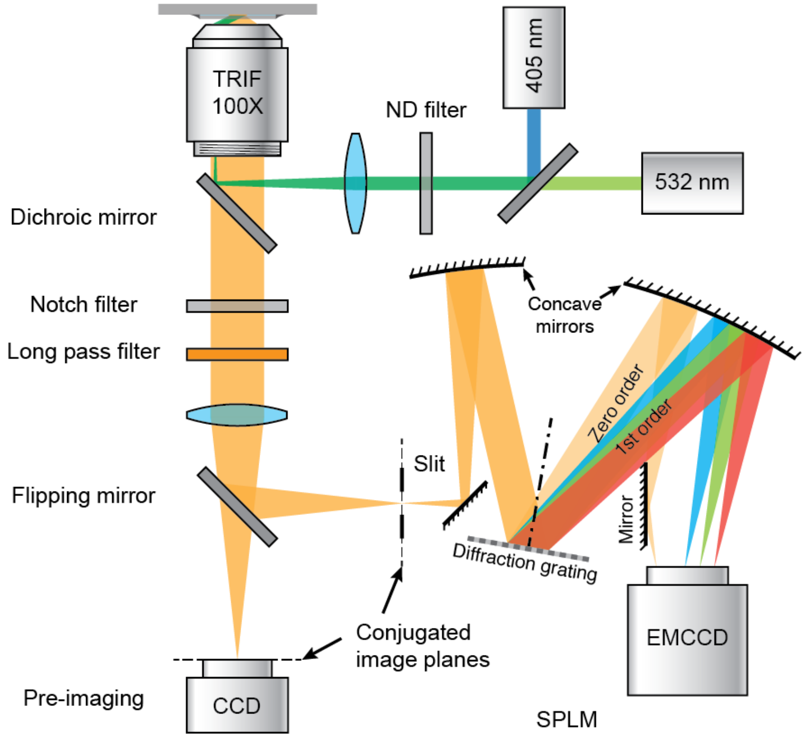

Supplementary Figure 1. The schematic of optical setup of SPLM. The excitation source used for demonstration was a 532 nm diode-pumped solid-state laser with 300-mW maximum output. After passing through a laser clean-up filter (LL01-532-12.5, Semrock) and further attenuating by a set of ND filters, it was coupled to an inverted microscope body (Nikon, Eclipse Ti-U), reflected off a high efficiency dichroic beamsplitter (LPD02-532RU-25, Semrock) and introduced into the sample through the back focal plane of a Nikon CFI apochromat TIRF objective lens (100X, 1.49 NA). By shifting the laser beam toward the edge of the TIRF objective with a translation stage, the emerging light reached the sample at the near critical angle of the glass-water interface, thus illuminating only the fluorophores within a controlled range (usually a micrometer) above the coverslip surface. A 532-nm notch filters (OD>6, NF01-532U-25, Semrock) and a long-pass filter (BLP01-532R-25, Semrock) were placed at the emission port to reject the reflected laser beam. A Czerny-Turner monochromator (SP2150, Princeton Instruments) with a grating (150 lines/mm) was placed at the conjugated image plane of the objective lens to disperse fluorescence emission from each diffraction-limited blinking.

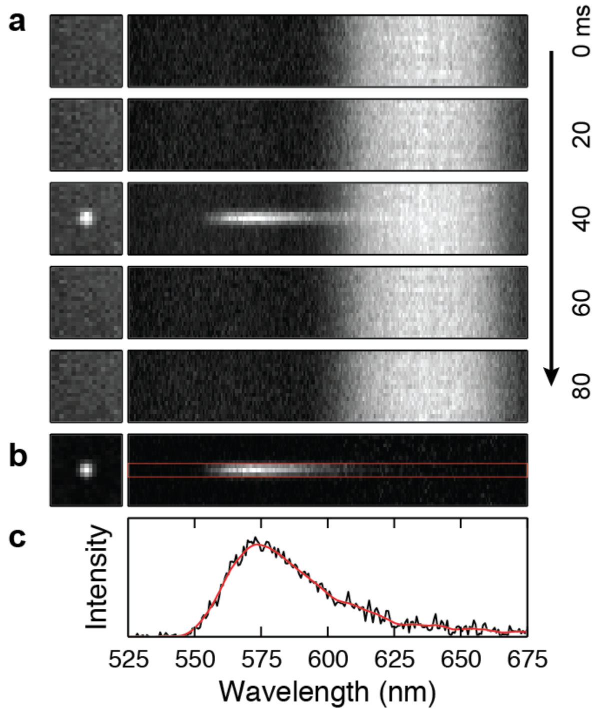

Supplementary Figure 2. Extracting spectrum of a single stochastic localization event. (a) Consecutive images of a single stochastic localization event in zero-order (left panel) and first-order (right panel), respectively. The bright band on the right is from Raman scattering of glass coverslip and solution. (b) Background was removed by subtracting the average of images without molecular emission from nearby frames. (c) After calibrating the pixel energy channels as shown in Supplementary Figure S3, spectrum was further divided by the wavelength dependent transmission and reflection of optical components and the camera quantum efficiency (Supplementary Figure 4) to recover the actual radiation spectrum.

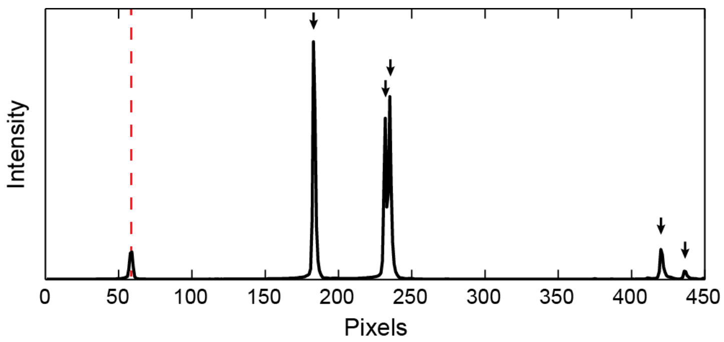

Supplementary Figure 3. The spectrum measured from a Mercury-Argon calibration lamp (SPL-HGAR, Photonics Technologies) was used to calibrate the pixel coordinate to wavelength. The red dashed line indicates the slit position. Emission peaks denoted by arrows from left to right are from 546.08 nm, 576.96 nm, 579.07 nm, 696.54 nm and 706.72 nm spectral lines, respectively.

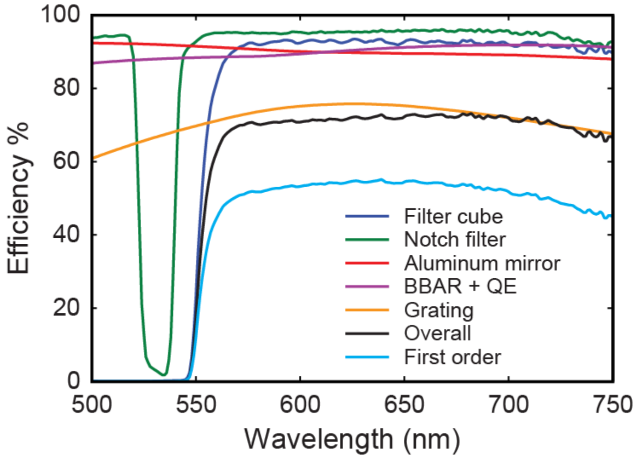

Supplementary Figure 4. Efficiency of the optical system. Blue line: transmission spectra of the combination of dichroic beamsplitter (LPD02-532RU-25, Semrock) and long pass filter (BLP01-532R-25, Semrock) in the filter cube. Green line: transmission spectra of the 532-nm notch filters (NF01-532U-25, Semrock). Red line: reflectance spectrum of aluminum mirrors. Purple line: the wavelength dependent quantum efficiency (QE) of the EMCCD with broadband anti-reflection (BBAR) coating. Orange line: the diffraction efficiency of the diffraction grating. Black line: the overall efficiency of the system. Cyan line: the overall efficiency of the first order.

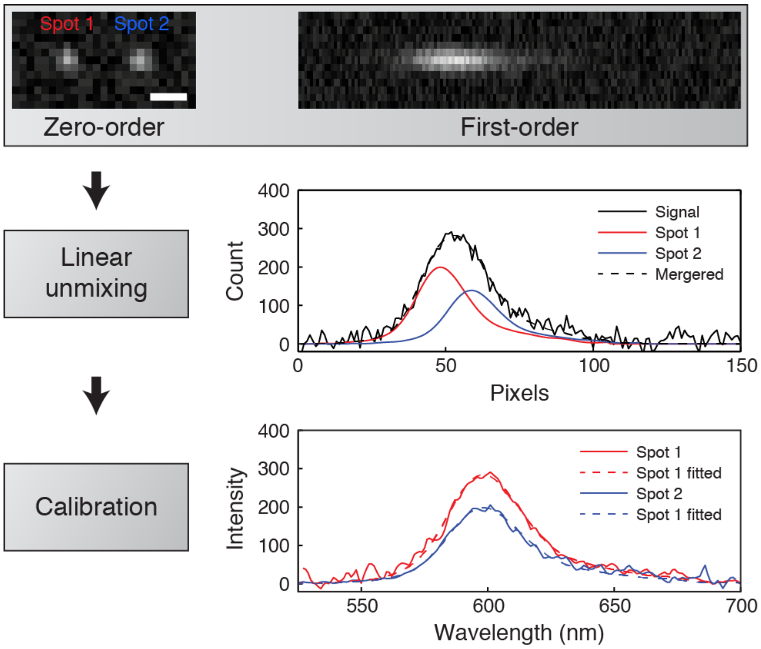

Supplementary Figure 5. Flow chart of spectral separation with modified linear unmixing algorithm. Scale bar: 500 nm.

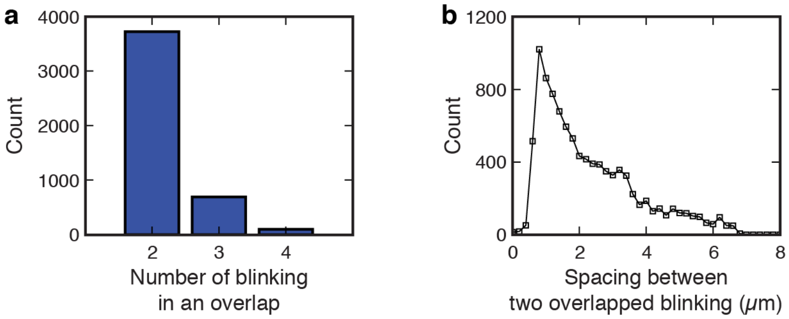

Supplementary Figure 6. Representative statistic results of spectral overlapping from 10,000 blinking acquired from the Rhodamine-labeled microtubule sample used in Figure 3. (a) Histogram of number of blinking in an overlap. (b) Histogram of spacing between two near-by overlapped blinking.

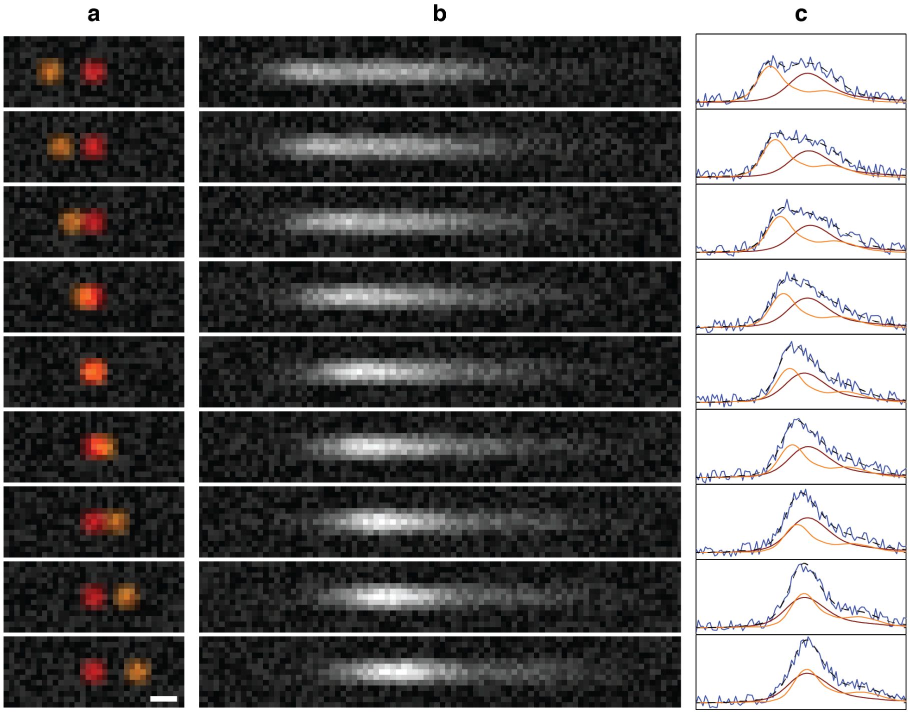

Supplementary Figure 7. Simulation of spectral separation for dual color imaging. (a) Two blinking at same horizontal position with spatial separation from -800 nm to 800 nm. Scale bar: 500 nm. (b) Simulation of the overlapped spectra. (c) Spectral separation with modified spectral unmixing algorithm.

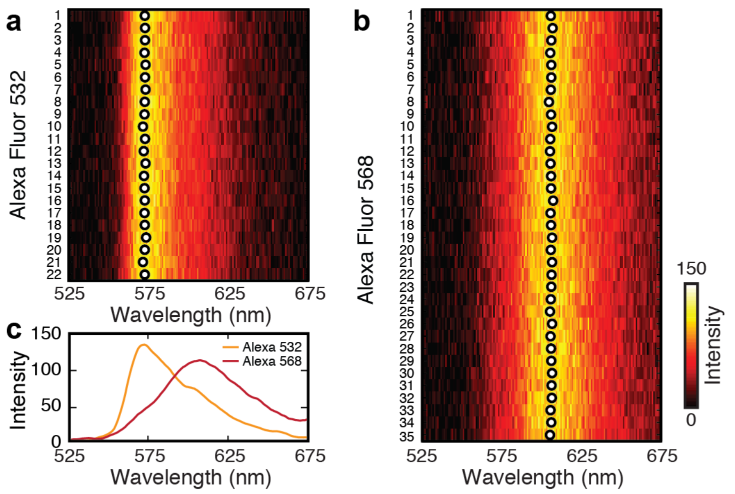

Supplementary Figure 8. Spectra of individual stochastic localizations were separated according to the emission characterization of (a) Alexa Fluor 532 and (b) Alexa Fluor 568, respectively. (c) Averaged emission spectra of two molecules.

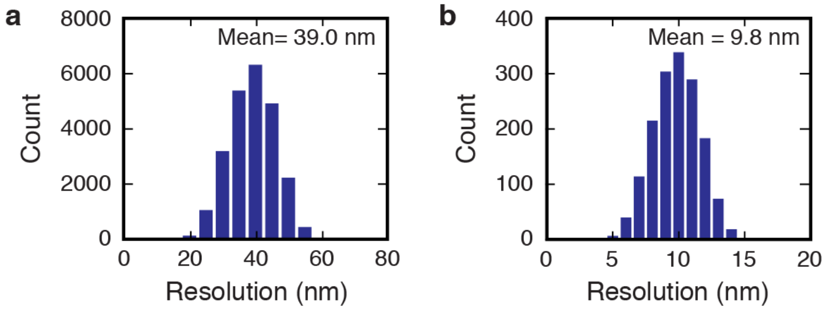

Supplementary Figure 9. Resolution analysis before (a) and after (b) spectral regression, respectively.

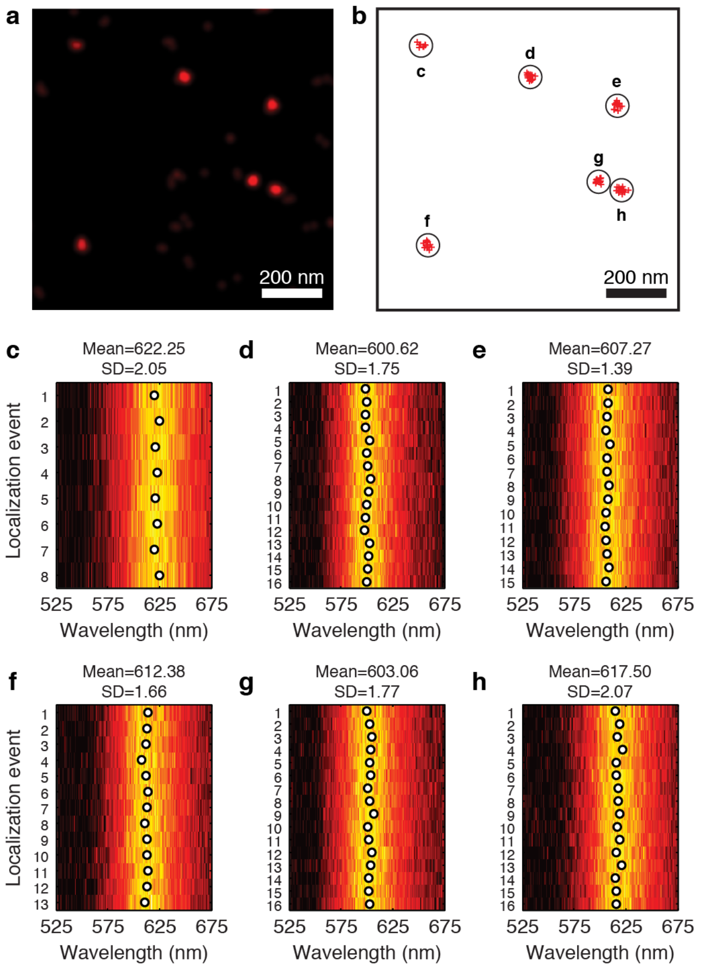

Supplementary Figure 10. (a) PLM image of actin monomers labeled by Alexa Fluor 568. (b) Centroids of localizations in clusters with more than 5 localizations. (c-h) Spectra of stochastic localizations denoted in (b), respectively.

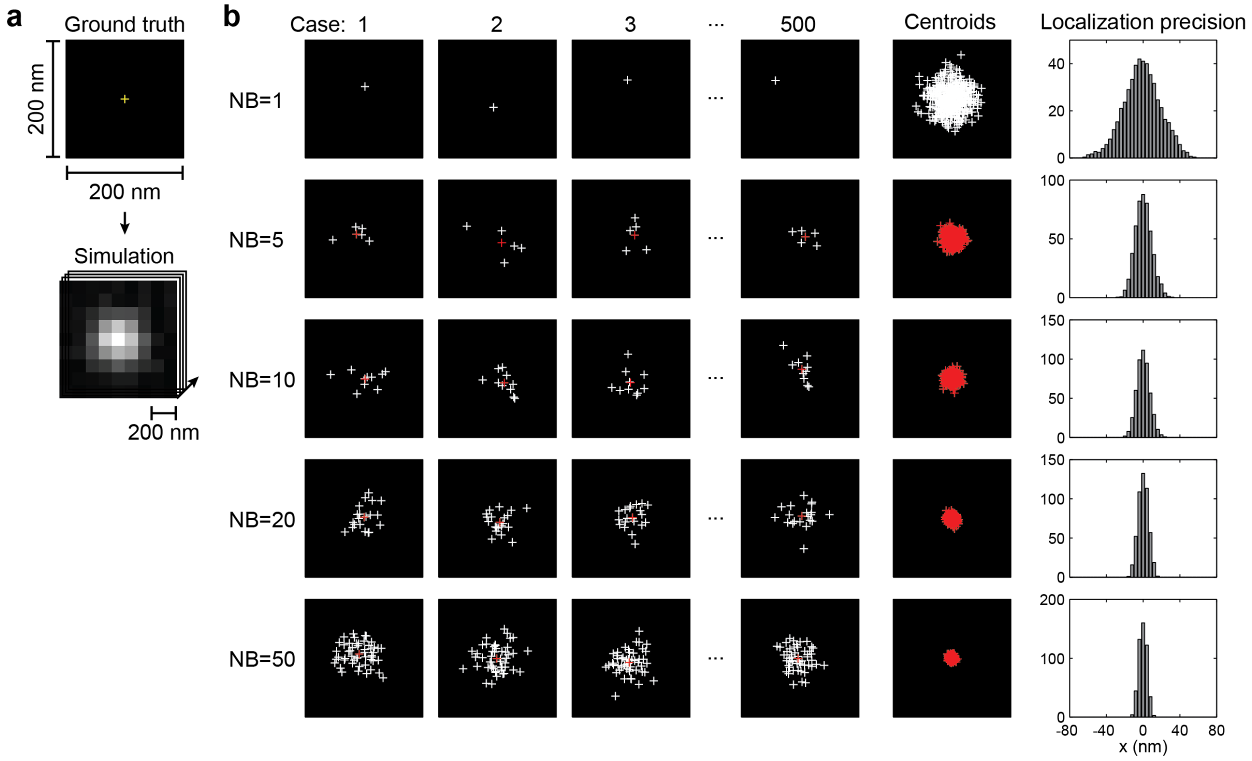

Supplementary Figure 11. (a) Ground truth of the molecule (upper panel) and a simulated single blinking event at diffraction-limited resolution (lower panel). (b) Improved resolution using molecular regression. White crosses denoted positions of all localizations in each test and red crosses denoted their centroids. Localization precision with respect to the number of blinking (*NB*) is calculated from the FWHM of the histogram, which are 37.4 nm, 16.5 nm, 11.7 nm, 7.9 nm and 5.1 nm for *NB*=1, 5, 10, 20 and 50, respectively.

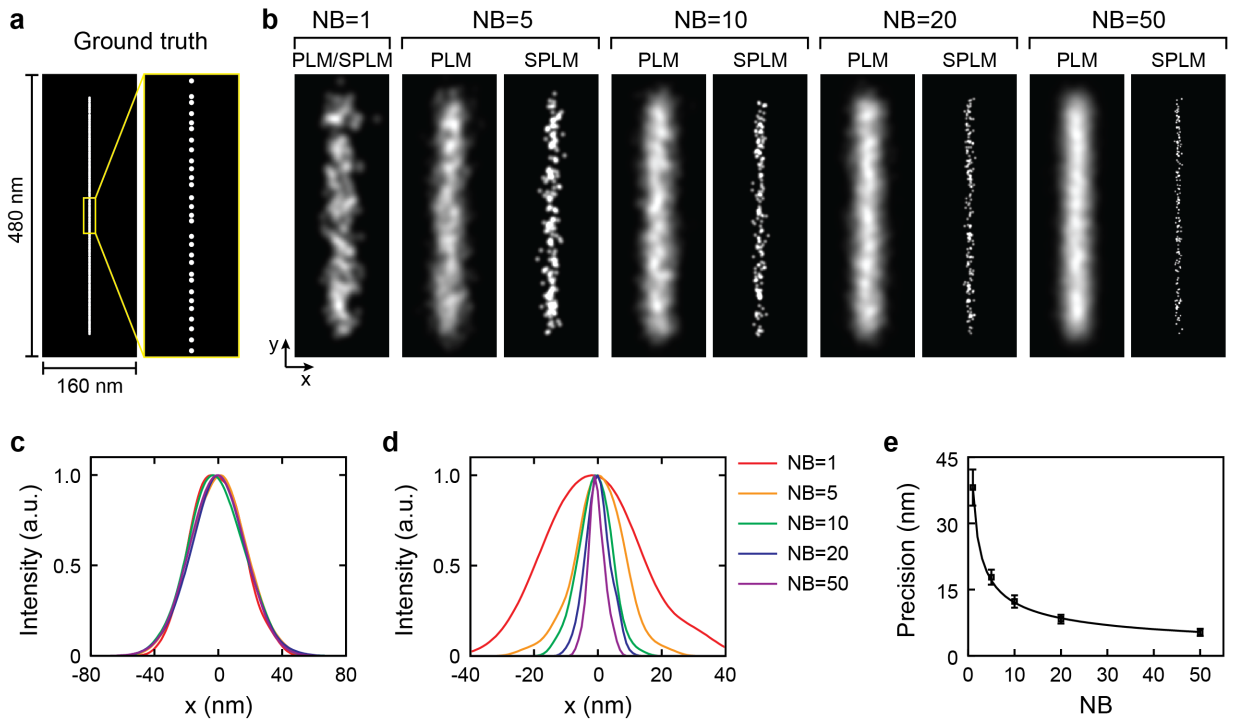

Supplementary Figure 12. (a) Ground truth of a random generated line pattern. The pattern was used to simulate a movie consisting of both zero-order and first order images for testing the performance of spectral regression method. The number of blinking (*NB*) from each molecule is set to be 1, 5, 10, 20 and 50, respectively. (b) PLM and SPLM images reconstructed from the simulated movie. Spectral regression was performed in SPLM reconstruction. Localization precision was calculated by average images along y-axis and was then normalized for comparison. (c) PLM images show same localization precision regardless the *NB*. (d) SPLM images have improved localization precision when *NB* increases. (e) Localization precision with respect to *NB* in SPLM. Black line is the curve calculated by $P_0/\sqrt{NB}$, where P_0 is the localization precision at *NB*=1.

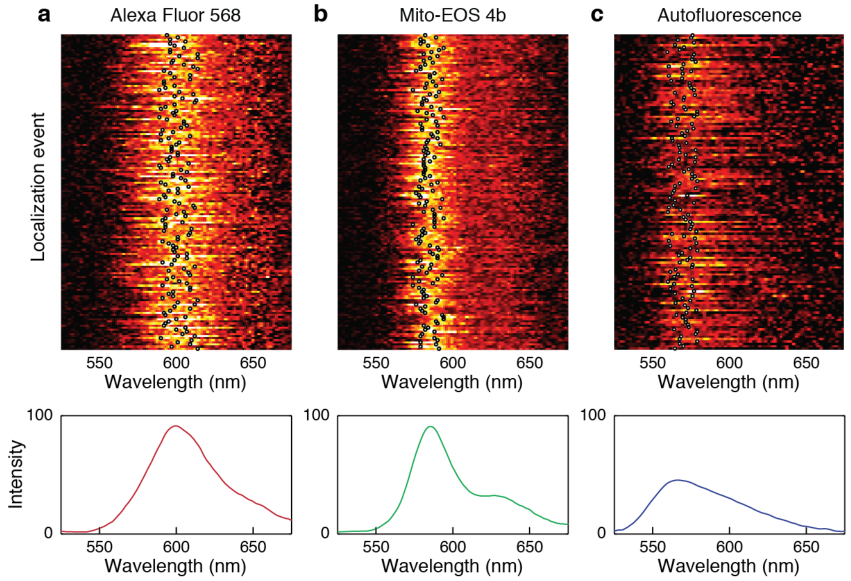

Supplementary Figure 13. Representative single-molecule spectra and their corresponding averaged spectra of (a) Alexa Fluor 568, (b) Mito-EOS 4b and (c) autofluorescence, respectively.

Supplementary Notes

Supplementary Note 1

Spectral unmixing

Stochastic blinking events occurring along the same horizontal position within the same frame can cause spectral overlap, which is highly probable in regions with many fluorophores in close proximity. Since the locations of blinking events are unambiguous in the direct image and the spectra from different blinking events are linearly mixed in the spectral image, overlapping can thus be separated with a modified spectral unmixing algorithm[1]. If we have n spectra from same type of dye molecule with emission spectrum s and the ith spectrum $s(x_i)$ emitted at x_i position with intensity of a_i, the observed spectrum S can be expressed as

$$S = \sum_{i=1}^{n} a_i\, s(x_i) + w, \tag{1}$$

where w is an error term accounting for additive noise (such as sensor noise and model inadequacies). Supplementary Figure 5 is the flow chart of spectral separation of two identical dye molecules. We simulated the spectral overlapping from two nearby molecules. Background noise was introduced in the image to mimic the SNR of actual fluorescence image. As an optimization process, we applied a linear least-squares solver, a built-in solver in MATLAB, to solve the above equation with a known number of localization events, where a_i and x_i are free parameters. After conducting unmixing, overlapped spectra can be separated as shown in Supplementary Figure 5. By using positions of two localization events as inherent reference points, we further calibrated their spectra from their pixel coordinates. Finally, spectra were divided by the wavelength dependent system efficiency to recover the actual emission spectra.

Acquiring accurate reference spectra is essential in generating satisfactory spectral unmixing results. Although the spectral profiles of synthetic dyes and fluorescent proteins are accurately known, they are usually measured from molecule assembles with spectral broadening due to underlying conformational heterogeneity. To obtain the reference spectra for SPLM, we measure the fluorescence spectrum from single molecule emission in the absence of inhomogeneous broadening. It can be easily obtained from frames with sparse single-molecule events. For single molecule spectroscopy, we also have to consider the spectral shift $\Delta\lambda_i$ from the underlying conformational heterogeneity. The observed spectrum S can be further expressed as

$$S = \sum_{i=1}^{n} a_i\, s(x_i + d_i) + w, \tag{2}$$

where d_i is corresponding shift in pixel domain, which can be calculated as $\Delta\lambda_i$/0.63nm.

In practical imaging, the blinking density needs to be carefully controlled to avoid a mass number of

overlapping spectra, while still maintaining sufficient number of localizations for achieving a satisfactory image quality[2]. Since the stochastic blinking events are also separated in time, mass overlap in single frames is rare. Typically, spectral overlap is from two or three molecules as shown in the statistic analysis in Supplementary Figure 6.

To verify the accuracy of our applied spectral unmixing algorithm in multicolor imaging, we simulated the situation with two types of dyes used in the cellular imaging, namely Alexa Fluor 568 and Mito-EOS 4b. For the case with more than one type of dye molecule, S can be simply expressed as

$$S = \sum_{i=1}^{n} a_i\, s_1(x_i + d_i) + \sum_{i=1}^{m} a_i\, s_2(x_i + d_i) + \cdots + w, \qquad (3)$$

wherer s_n is the emission spectrum of type n dye molecule. As shown in Supplementary Figure 7, two blinking events with separation from 0 nm to ±800 nm were used to simulate overlapped spectra in the spectral image. The simulation shows a satisfied result of spectral separation with our modified unmixing algorithm.

Supplementary Note 2

Improving localization precision via spectral regression

The precision of identifying the centroid location (σ) can be approximated by the probability equation[2]

$$\sigma = \sqrt{\left(\frac{s_i^2}{N}\right) + \left(\frac{a^2/12}{N}\right) + \left(\frac{8\pi s_i^4 b^2}{a^2 N^2}\right)}, \qquad (4)$$

where s_i is the standard deviation of the Gaussian fit in x and y direction; N is the number of detected photons; a is the pixel size of the CCD camera; and b is the standard deviation of the CCD background. As we can see, the localization uncertainty is proportional to the inverse square root of the number of detected photons.

To further validate the resolution improvement with spectral regression, we first performed a simulation in the case of a single molecule and calculated the precision after regression, as shown in Supplementary Figure 11. In general, the regression can be judged by any molecular specific parameter, such as intensity, polarization, anisotropy, and emission spectrum. Since we are considering a single molecule case in this simulation, all blinking events being collected are naturally from the same molecule and no regression is necessary. The feasibility of our spectral regression algorithm will be discussed in the next case of simulation using the line pattern.

In order to examine the localization precision with respect to the number of blinking (NB) from each molecule, we first generated a movie that consisted of NB frames with a pixel size of 100 nm. In each frame, a single blinking event at diffraction-limited resolution (predefined by the objective NA at

wavelength of 600 nm) was superimposed on a Gaussian noise background (Supplementary Figure 11a). The total photon count and noise level were adjusted to match our experimental conditions. After reconstruction using a standard PALM/STORM algorithm, positions of all the collected localizations were plotted as white crosses. Their centroid, which represents the result of the regression, was further plotted as a red cross. As shown in Supplementary Figure 11b, we tested 500 randomly generated cases to evaluate the localization precision of centroids by plotting the histogram along one of the lateral directions (x-axis). When NB=1, the result shows the precision of conventional PLM (37.4 nm) since no regression was used. As NB increases, the localization precision improves, reaching a resolution to 5.1 nm (7.3-fold improvement) when NB=50. In practice, the NB acquired from experiments normally ranges between 10 and 20. Thus the experimental resolution we can typically achieve is between 7.9 nm and 11.7 nm. To achieve even higher resolution, we can prolong image acquisition time and acquire more blinking events from the same molecules if they still generate blinking. These results illustrate the principle of using spectral regression to improve image resolution in SPLM.

Since spectroscopic information provides one of the most important evidence for molecular discrimination, we hereby used SPLM to realize spectral regression to improve spatial resolution. To demonstrate the feasibility and the performance of our spectral regression method for densely labeled molecules, we performed a simulation using a straight-line pattern consisting of randomly arranged molecules with line density of 1 nm^{-1}. Supplementary Figure 12a shows the ground truth of the line pattern. To generate the simulated movie for spectral regression of a single molecule type, the emission spectrum of each molecule was modeled after the emission properties of Alexa Fluor 568, where the peak emission is 610 nm. A fixed wavelength shift in the range of ±11 nm was further assigned to the modeled spectrum, which represents the spectral variation of Alexa Fluor 568 observed in the single molecule experiment shown in Supplementary Figure 10. In the generated movie, each frame contains one stochastic blinking event in the zeroth order and a spectrum in the first order images, which are similar to the simulated image shown in Supplementary Figure 7. All simulation conditions, including sizes of the pixel and the point spread function, photon number and background noise level, were set to be exactly the same as the previous simulation for a fair comparison. We then performed reconstruction and collected locations of each blinking event and the associated spectra. The spectral shift of each spectrum $\Delta\lambda_i$ was calculated based on the shift of the spectral centroid by using the following equation:

$$\Delta\lambda_i = \frac{\sum_\lambda \lambda I(\lambda)}{\sum_\lambda I(\lambda)} - \Delta\lambda_0, \quad (5)$$

where λ is the wavelength; $I(\lambda)$ represents the spectral intensity at λ; and $\Delta\lambda_0$ is the centroid of the un-shifted spectrum.

To perform the appropriate spectral regression, we have to establish reasonable criteria. First, only

localizations within the range of localization precision in the zeroth-order image (37.4 nm) were considered. The criteria in the spectral domain were derived from the experimental single molecule spectroscopy data acquired by SPLM simultaneously, as shown in Figure 3e. The spectral shift from the same molecule should be less than 2 nm, which was derived from the maximum standard deviation of spectral variation from the single molecule experiment (Supplementary Figure 10). Based on experimental observation, an acceptable variation of intensity was set to be ±10% of the integrated intensity of the spectrum. All localizations were then judged by these criteria to be clustered and merged as molecules.

Supplementary Figure 12b shows the reconstructed images of PLM and SPLM, where only SPLM uses the spectral regression as mentioned above. Resolution improvement can be clearly observed when NB increases. This is consistent with the previous simulation performed in a single molecule situation. Additionally, we calculated the resulting resolution of SPLM as a function of NB by averaging images along the vertical direction (y-axis), as shown in Supplementary Figure 12c. In conventional PLM without regression, the resolution obtained after reconstruction is independent of the NB, remaining identical as 38.1±4.1 nm. In Supplementary Figure 12d, the resolutions of SPLM are respectively improved to be 17.8±1.7 nm, 12.3±1.4 nm, 8.3±1.0 nm and 5.3±0.8 nm when NBs from each molecule are 1, 5, 10, 20 and 50. They follow the trend of $P_0/\sqrt{NB}$, where P_0 is the localization precision when NB=1, as shown in Supplementary Figure 12e. This trend suggests that spectral regression indeed improves the localization precision by increasing the photon counts for localization analysis. Even with the strict rejection criteria descried above, it's worth mentioning that 90±5% and 85±6% of localizations for the individual molecules can be recovered when NB=10 and NB=20, respectively, when compared with the ground truth. This demonstrates the feasibility of spectral regression to be performed in densely labeled samples and it sets the foundation of image reconstruction in Figure 3 and 4.

For multi-labeled sample, we first classified localizations by molecule types based on their spectra and then applied separate criteria in spectral regression for molecules of different types based on the analysis of data shown in Supplementary Figure 13.

References:

1 Zimmermann, T. Spectral imaging and linear unmixing in light microscopy. *Adv Biochem Eng Biot* **95**, 245-265 (2005).

2 Dempsey, G. T., Vaughan, J. C., Chen, K. H., Bates, M. & Zhuang, X. W. Evaluation of fluorophores for optimal performance in localization-based super-resolution imaging. *Nat Methods* **8**, 1027 (2011).
